# Supplementary figures and images for: Identification and pathogenicity of Alternaria species associated with leaf blotch disease and premature defoliation in French apple orchards
Source: PeerJ. 2021 Dec 1;9:e12496. doi: 10.7717/peerj.12496 (PMC8643104; doi:10.7717/peerj.12496)

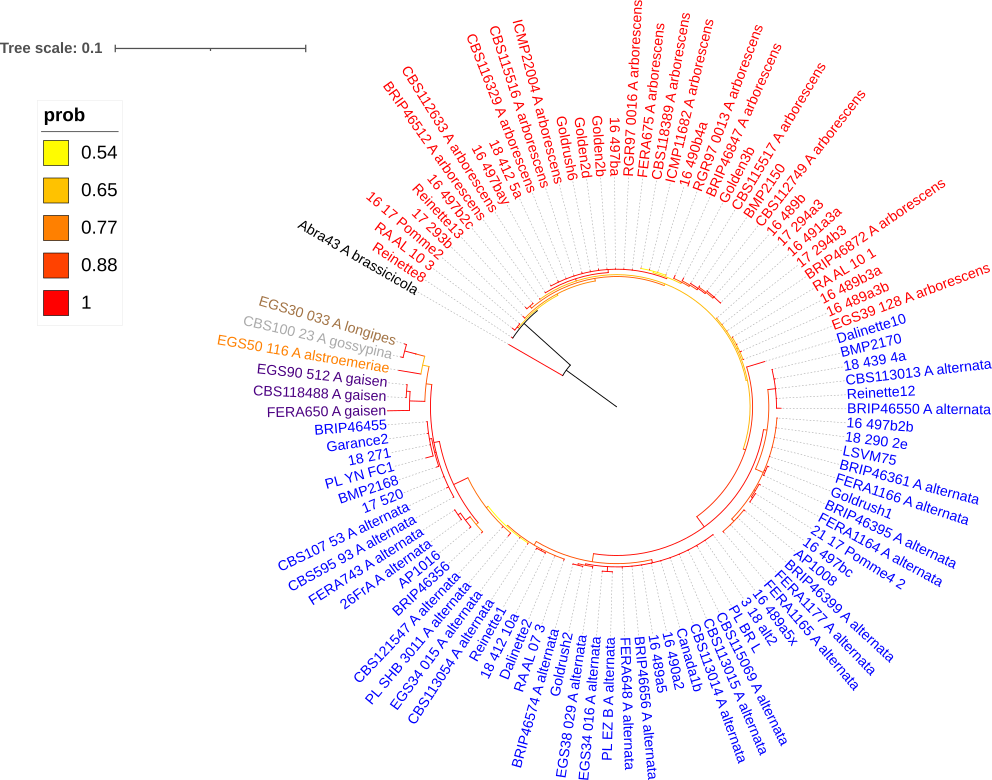

Supplement: Supplemental Information 2 — The tree was constructed with sequences of 100 Alternaria isolates. The color legend refers to the Bayesian posterior probabilities of the tree nodes. Alternaria alternata isolate names are shown in blue. Alternaria arborescens SC isolates are shown in red. Isolates from other taxonomic groups of the Alternaria section Alternaria are represented in orange (Alternaria alstroemeriae), purple (Alternaria gaisen), brown (Alternaria longipes) and grey (Alternaria gossypina). [file peerj-09-12496-s002.png]

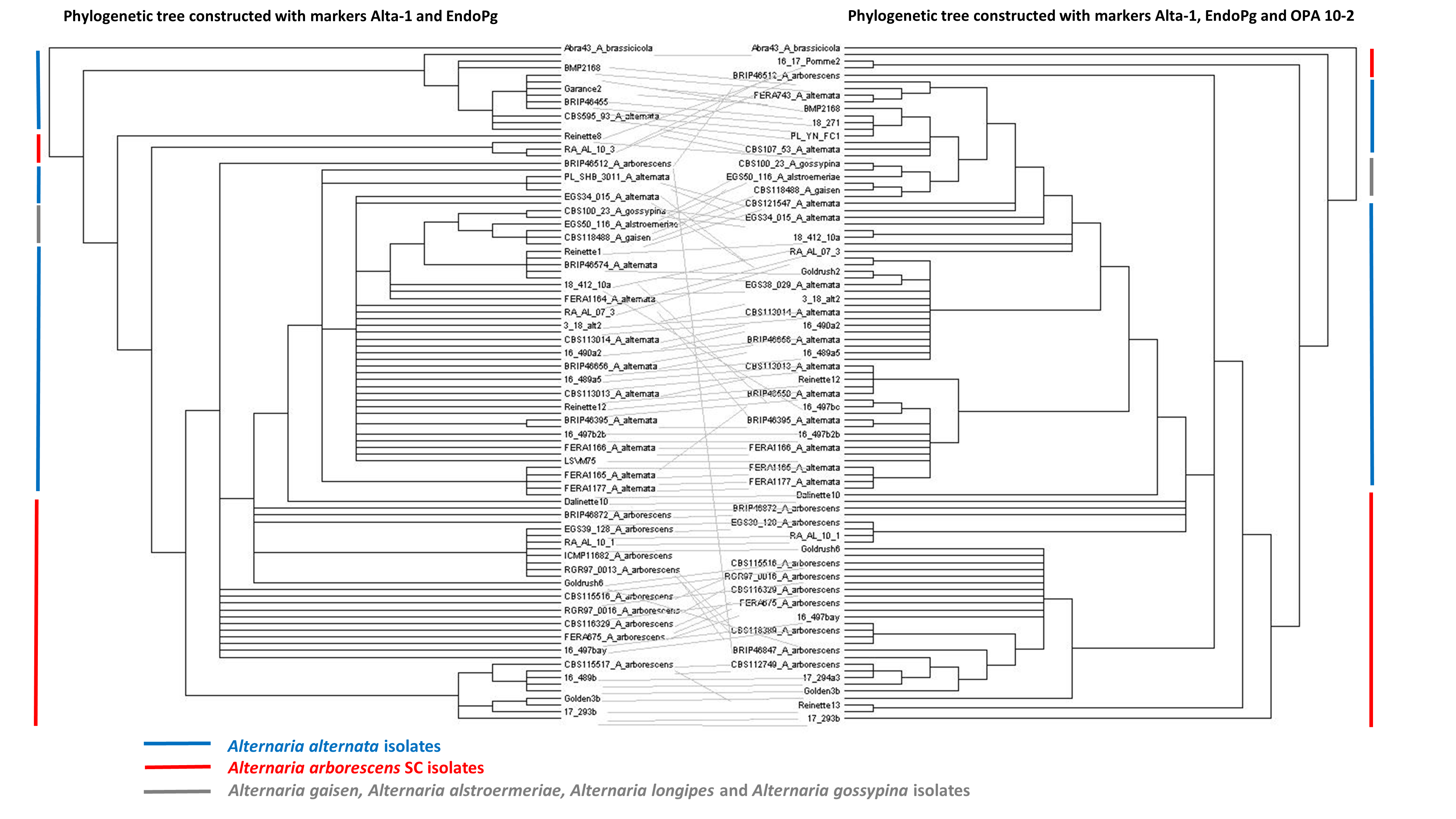

Supplement: Supplemental Information 3 — The analysis included a subset of 100 isolates. Isolate clades are represented by different colors: blue for Alternaria alternata, red for Alternaria arborescens SC and grey for Alternaria gaisen, Alternaria alstroemeriae, Alternaria longipes and Alternaria gossypina. The figure only shows some of the isolate names. [file peerj-09-12496-s003.png]
