# Supplementary material for: Identification and pathogenicity of Alternaria species associated with leaf blotch disease and premature defoliation in French apple orchards
Source: PeerJ. 2021 Dec 1;9:e12496. doi: 10.7717/peerj.12496 (PMC8643104; doi:10.7717/peerj.12496)
Supplement: Supplemental Information 5 — Inoculation tests with detached leaves of the Gala apple cultivar. The codes next to each leaf are isolate codes. Isolates of Alternaria arborescens are shown in red and isolates of Alternaria alternata in purple. (B) Inoculation tests with detached leaves of the Golden Delicious apple cultivar. The codes next to each leaf are isolate codes. Isolates of Alternaria arborescens are shown in red and isolates of Alternaria alternata in purple. Pathogenicity tests were performed by inoculation of unwounded abaxial leaf surfaces with 10 µL of conidial suspensions (concentration of 1 × 10^5^ conidia/mL). An experimental replicate consisted of one strain inoculated on five different leaves (placed in five different plastic boxes) per cultivar. [file peerj-09-12496-s005.docx]

Examples of a single experimental replicate used in pathogenicity tests 4, 7 and 10 days post inoculation (dpi).

1. Inoculation tests with detached leaves of the Gala apple cultivar. The codes next to each leaf are isolate codes. Isolates of *Alternaria arborescens* are shown in red and isolates of *Alternaria alternata* in purple.
2. Inoculation tests with detached leaves of the Golden Delicious apple cultivar. The codes next to each leaf are isolate codes. Isolates of *Alternaria arborescens* are shown in red and isolates of *Alternaria alternata* in purple.

Pathogenicity tests were performed by inoculation of unwounded abaxial leaf surfaces with 10 µL of conidial suspensions (concentration of 1x10^5^ conidia/mL). An experimental replicate consisted of one strain inoculated on five different leaves (placed in five different plastic boxes) per cultivar.

1.
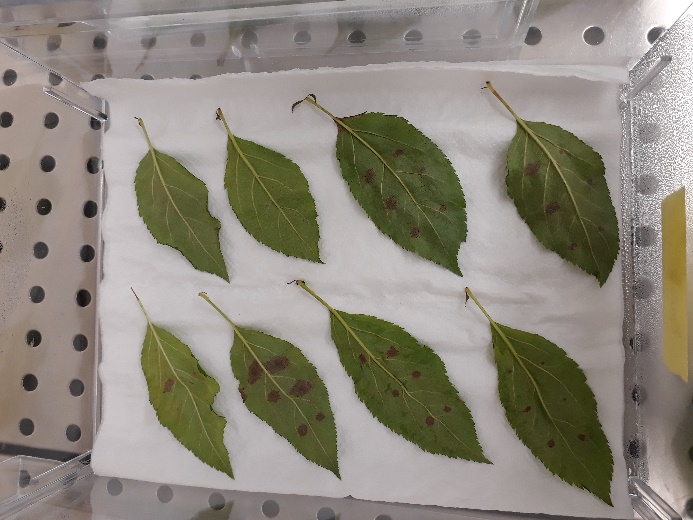

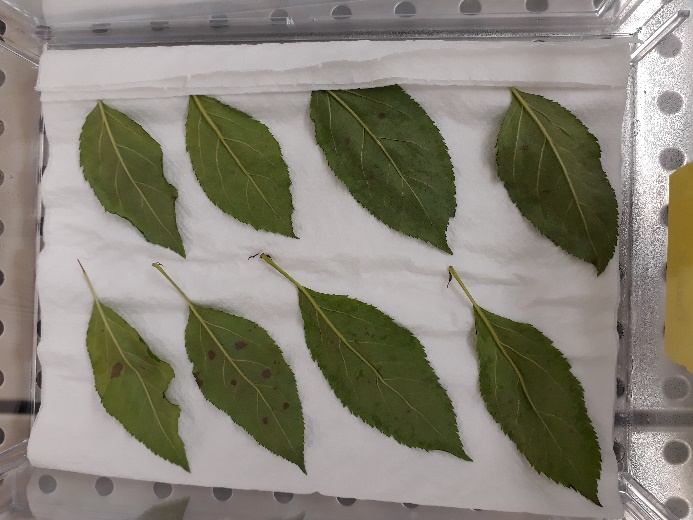

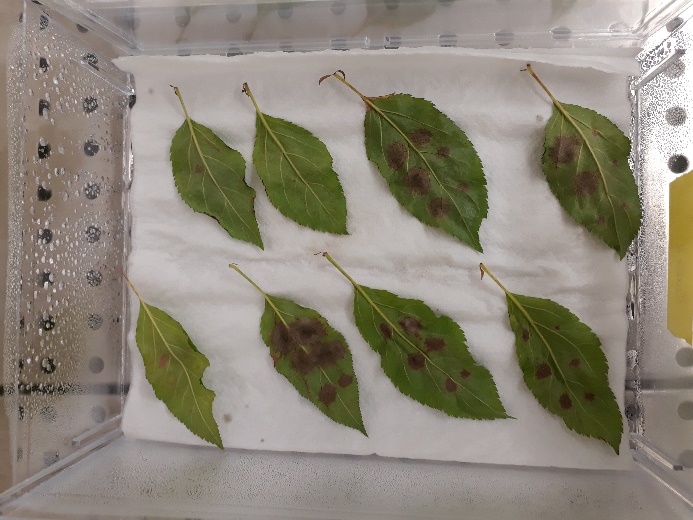
Gala cultivar

**LSVM1443**

**16_489a5x**

**16_489b1a**

**LSVM75**

7 dpi

**16_489b3a**

**16_490b4a**

**16_497bc**

Water control

10 dpi

4 dpi

**16_489b3a**

**16_490b4a**

**16_497bc**

Water control

**LSVM1443**

**16_489a5x**

**16_489b1a**

**LSVM75**

**LSVM1443**

**16_489a5x**

**16_489b1a**

**16_489b3a**

**16_490b4a**

**16_497bc**

Water control

**LSVM75**

1.
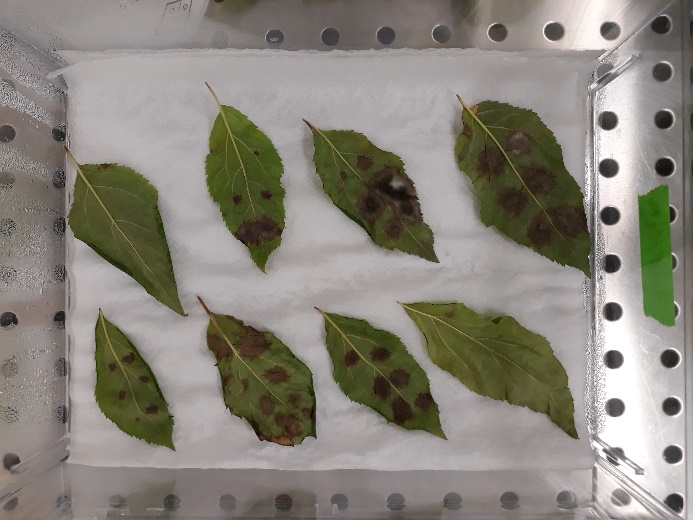

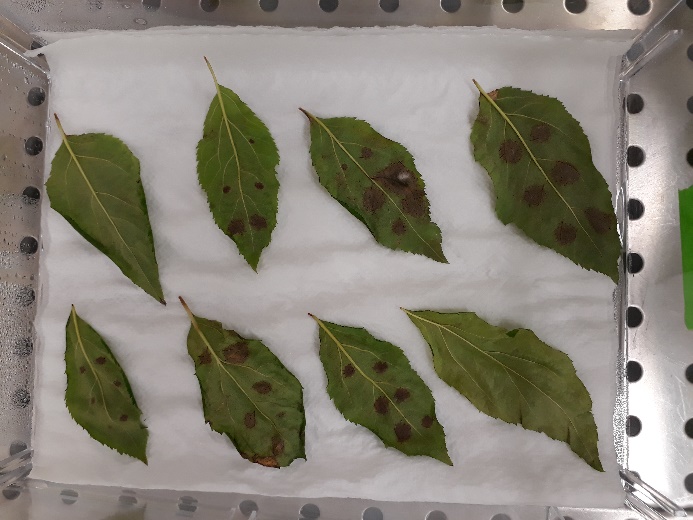

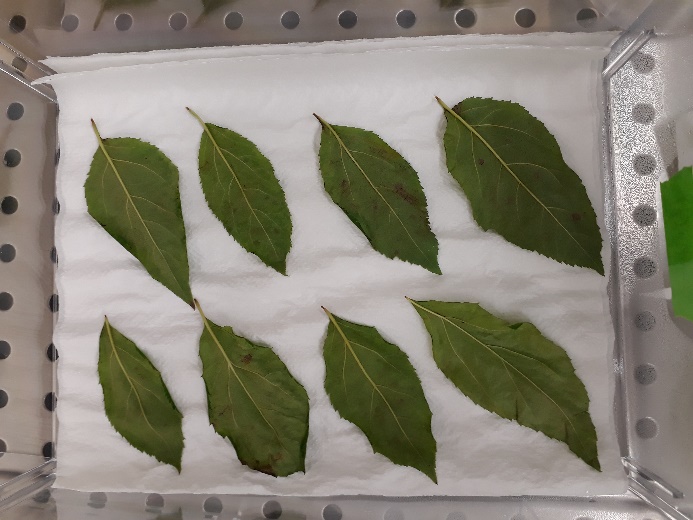
Golden Delicious cultivar

7 dpi

10 dpi

**PL_Nov_Li**

**LSVM1451**

**FERA648**

**17_520**

**16_491a3a**

Water control

**21_17_Pomme4_2**

**PL_Nov_Li**

**LSVM1451**

**FERA648**

**21_17_Pomme4_2**

**17_520**

**16_491a3a**

**Golden D. 2b**

Water control

**PL_Nov_Li**

**LSVM1451**

**FERA648**

**21_17_Pomme4_2**

**17_520**

**16_491a3a**

**Golden D. 2b**

Water control

4 dpi
